# Tetra­ethyl­ammonium 4-hy­droxy­benzoate monohydrate

**DOI:** 10.1107/S1600536811024044

**Published:** 2011-06-25

**Authors:** Heping Li, Pei Liu, Yunxia Yang

**Affiliations:** a Henan University of Traditional Chinese Medicine, Zhengzhou 450008, People’s Republic of China; b Key Laboratory of Polymer Materials of Gansu Province, Ministry of Education, College of Chemistry and Chemical Engineering, Northwest Normal University, Lanzhou 730070, Gansu, People’s Republic of China

## Abstract

In the title compound, C_8_H_20_N^+^·C_7_H_5_O_3_^−^·H_2_O, the carboxyl­ate group is slightly out of the plane of the parent benzene ring, the C—C—C—O torsion angles being 2.3 (2) and 2.0 (2)°. The carboxyl­ate group and the hy­droxy group form O—H⋯O hydrogen bonds, generating a head-to-tail chain along the *b* axis. Neighbouring hydrogen-bonded chains are linked by the water mol­ecule, generating two independent O—H⋯O donor hydrogen bonds. The carboxyl­ate group thus constructs a hydrogen-bonded host layer parallel to (10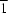). The tetra­ethyl­ammonium cation is contained between these layers, forming a sandwich-like structure with an approximate inter­layer distance of 10.03 Å.

## Related literature

*p*-Hy­droxy­benzoic acid has been found to inter­act with varied cations, such as dec­yl(trimeth­yl)ammonium and hexa­methonium, to form different crystal structures, see: Marsh & Spek (2001 ▶); Yang *et al.* (2010 ▶).
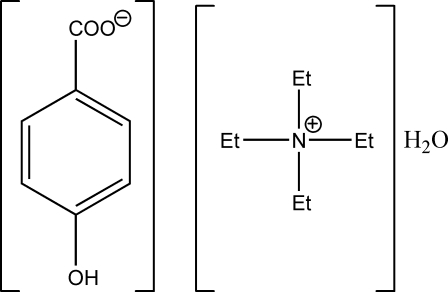


## Experimental

### 

#### Crystal data

C_8_H_20_N^+^·C_7_H_5_O_3_^−^·H_2_O
*M* *_r_* = 285.38
Monoclinic, 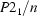

*a* = 9.6082 (10) Å
*b* = 16.2610 (16) Å
*c* = 10.4478 (10) Å
β = 96.378 (1)°
*V* = 1622.2 (3) Å^3^
*Z* = 4
Mo *K*α radiation
μ = 0.08 mm^−1^
*T* = 296 K
0.66 × 0.37 × 0.20 mm

#### Data collection

Bruker SMART APEX diffractometer
Absorption correction: multi-scan (*SADABS*; Sheldrick, 1996 ▶) *T*_min_ = 0.947, *T*_max_ = 0.984
7411 measured reflections
3774 independent reflections
2730 reflections with *I* > 2σ(*I*)
*R*_int_ = 0.015

#### Refinement

*R*[*F* ^2^ > 2σ(*F* ^2^)] = 0.055
*wR*(*F* ^2^) = 0.181
*S* = 1.06
3774 reflections
182 parameters
4 restraints
H-atom parameters constrained
Δρ_max_ = 0.35 e Å^−3^
Δρ_min_ = −0.19 e Å^−3^

### 

Data collection: *APEX2* (Bruker, 2007 ▶); cell refinement: *SAINT* (Bruker, 2007 ▶); data reduction: *SAINT*; program(s) used to solve structure: *SHELXS97* (Sheldrick, 2008 ▶); program(s) used to refine structure: *SHELXL97* (Sheldrick, 2008 ▶); molecular graphics: *SHELXTL* (Sheldrick, 2008 ▶); software used to prepare material for publication: *SHELXL97* and *publCIF* (Westrip, 2010 ▶).

## Supplementary Material

Crystal structure: contains datablock(s) I, global. DOI: 10.1107/S1600536811024044/fj2433sup1.cif

Structure factors: contains datablock(s) I. DOI: 10.1107/S1600536811024044/fj2433Isup2.hkl

Supplementary material file. DOI: 10.1107/S1600536811024044/fj2433Isup3.cml

Additional supplementary materials:  crystallographic information; 3D view; checkCIF report

## Figures and Tables

**Table 1:** Hydrogen-bond geometry (Å, °)

*D*—H⋯*A*	*D*—H	H⋯*A*	*D*⋯*A*	*D*—H⋯*A*
O1—H1*A*⋯O3^i^	0.86	1.74	2.5984 (16)	175
O1*W*—H1*WA*⋯O3^ii^	0.85	2.04	2.850 (2)	161
O1*W*—H1*WB*⋯O2^iii^	0.85	1.94	2.781 (2)	169

Symmetry codes: (i) 
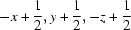
; (ii) 
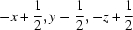
; (iii) 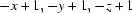.

**Fig. 1.**
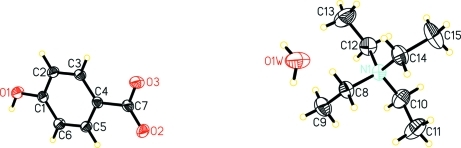
Thermal ellipsoid plot of the title compound at the 30% probability level; hydrogen atoms are drawn as spheres of arbitrary radius.

**Fig. 2.**
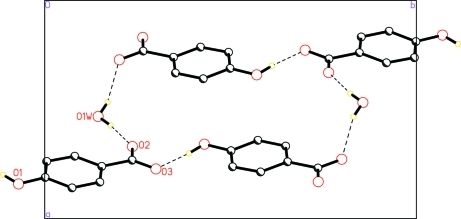
Hydrogen-bonded linking pattern of the host layer in the crystal structure of the title compound.

**Fig. 3.**
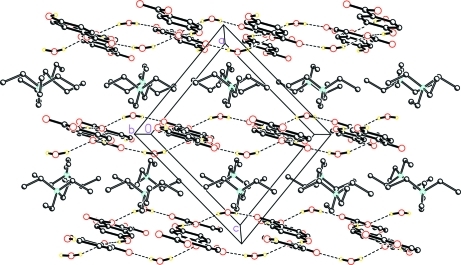
Packing diagram of the title compound; all hydrogen atoms bonded to carbon are omitted for clarity and the cations are represented with the open bonds.

## supplementary crystallographic information

### Comment

*p*-Hydroxybenzoic acid has been found to interact with varied cations, such as decyl(trimethyl)ammonium and hexamethonium, to form different crystal structures (Marsh *et al.*, 2001; Yang *et al.*, 2010). In the asymmetric unit of the title compound, (C_2_H_5_)_4_N^+^.C_7_H_5_O_3_^-^.H_2_O, there exist one *p*-hydroxybenzoate anion, in which the carboxyl group distorts a small angle with respect to the phenyl ring which has a mean deviation from plane of 0.0041 Å (the related torsion angles are 2.3 (2)° and 2.0 (2)° respectively), one water molecule and one tetraethylammonium cation (Fig. 1). With the help of the water molecule, the hydrogen-bonded chains of *p*-hydroxybenzoate anions extending along the [010] direction are connected with various O—H···O interactions to generate the hydrogen-bonded host layers (Fig. 2), which are parallel to the (101) plane and can accommodate the guest species of tetraethylammonium cations to form the final packing structure (Fig. 3). Obviously, water molecule, as a compensate host molecule, plays an important role in generating the hydrogen-bonded layer structure.

For the related crystal structures of *p*-hydroxybenzoic acid and different cations, see: Marsh *et al.*, (2001), Yang *et al.*, (2010).

### Experimental

*p*-Hydroxybenzoic acid (0.25 mmol, 0.035 g) was dissolved in small amount of water-ethanol (50:100 *v*/*v*) mixture and a 25% aqueous solution of tetraethylammonium hydroxide was added to neutralize the acid. Colorless block crystals separated after several weeks.

### Refinement

All non-hydrogen atoms were refined with anisotropic displacement parameters, and all the hydrogen atoms bonded to carbon were introduced into idealized dispositions. And the hydrogen atoms bonded to oxygen atoms were placed in difference map with fixed distance of 0.86 Å.

### Crystal data

**Table:**

C_8_H_20_N^+^·C_7_H_5_O_3_^−^·H_2_O	*F*(000) = 624
*M**_r_* = 285.38	*D*_x_ = 1.168 Mg m^−^^3^
Monoclinic, *P*2_1_/*n*	Mo *K*α radiation, λ = 0.71073 Å
Hall symbol: -P 2yn	Cell parameters from 2665 reflections
*a* = 9.6082 (10) Å	θ = 3.1–27.5°
*b* = 16.2610 (16) Å	µ = 0.08 mm^−^^1^
*c* = 10.4478 (10) Å	*T* = 296 K
β = 96.378 (1)°	Block, colorless
*V* = 1622.2 (3) Å^3^	0.66 × 0.37 × 0.20 mm
*Z* = 4	

### Data collection

**Table:**

Bruker SMART APEX diffractometer	3774 independent reflections
Radiation source: fine-focus sealed tube	2730 reflections with *I* > 2σ(*I*)
graphite	*R*_int_ = 0.015
φ and ω scans	θ_max_ = 27.7°, θ_min_ = 3.1°
Absorption correction: multi-scan (*SADABS*; Sheldrick, 1996)	*h* = −8→12
*T*_min_ = 0.947, *T*_max_ = 0.984	*k* = −21→16
7411 measured reflections	*l* = −12→13

### Refinement

**Table:**

Refinement on *F*^2^	Secondary atom site location: difference Fourier map
Least-squares matrix: full	Hydrogen site location: inferred from neighbouring sites
*R*[*F*^2^ > 2σ(*F*^2^)] = 0.055	H-atom parameters constrained
*wR*(*F*^2^) = 0.181	*w* = 1/[σ^2^(*F*_o_^2^) + (0.0974*P*)^2^ + 0.3052*P*] where *P* = (*F*_o_^2^ + 2*F*_c_^2^)/3
*S* = 1.06	(Δ/σ)_max_ = 0.001
3774 reflections	Δρ_max_ = 0.35 e Å^−^^3^
182 parameters	Δρ_min_ = −0.19 e Å^−^^3^
4 restraints	Extinction correction: *SHELXL97* (Sheldrick, 2008), Fc^*^=kFc[1+0.001xFc^2^λ^3^/sin(2θ)]^-1/4^
Primary atom site location: structure-invariant direct methods	Extinction coefficient: 0.011 (3)

### Special details

**Table:**

Geometry. All e.s.d.'s (except the e.s.d. in the dihedral angle between two l.s. planes) are estimated using the full covariance matrix. The cell e.s.d.'s are taken into account individually in the estimation of e.s.d.'s in distances, angles and torsion angles; correlations between e.s.d.'s in cell parameters are only used when they are defined by crystal symmetry. An approximate (isotropic) treatment of cell e.s.d.'s is used for estimating e.s.d.'s involving l.s. planes.
Refinement. Refinement of *F*^2^ against ALL reflections. The weighted *R*-factor *wR* and goodness of fit *S* are based on *F*^2^, conventional *R*-factors *R* are based on *F*, with *F* set to zero for negative *F*^2^. The threshold expression of *F*^2^ > σ(*F*^2^) is used only for calculating *R*-factors(gt) *etc*. and is not relevant to the choice of reflections for refinement. *R*-factors based on *F*^2^ are statistically about twice as large as those based on *F*, and *R*- factors based on ALL data will be even larger.

### Fractional atomic coordinates and isotropic or equivalent isotropic displacement parameters (Å^2^)

**Table:**

	*x*	*y*	*z*	*U*_iso_*/*U*_eq_	
C1	0.18857 (16)	0.99878 (8)	0.16322 (14)	0.0403 (3)	
C2	0.14101 (18)	0.92648 (9)	0.10187 (15)	0.0455 (4)	
H2A	0.0914	0.9286	0.0203	0.055*	
C3	0.16740 (16)	0.85171 (9)	0.16210 (15)	0.0418 (4)	
H3A	0.1363	0.8037	0.1197	0.050*	
C4	0.23956 (15)	0.84650 (9)	0.28496 (14)	0.0378 (3)	
C5	0.28608 (17)	0.91927 (9)	0.34473 (14)	0.0425 (4)	
H5A	0.3344	0.9173	0.4269	0.051*	
C6	0.26236 (17)	0.99454 (9)	0.28514 (15)	0.0432 (4)	
H6A	0.2958	1.0424	0.3266	0.052*	
C7	0.26794 (17)	0.76486 (9)	0.35068 (16)	0.0459 (4)	
C8	0.3736 (2)	0.18451 (12)	0.6489 (2)	0.0616 (5)	
H8A	0.4666	0.1638	0.6772	0.074*	
H8B	0.3597	0.1803	0.5558	0.074*	
C9	0.3679 (3)	0.27388 (14)	0.6854 (3)	0.0901 (8)	
H9A	0.4380	0.3038	0.6459	0.135*	
H9B	0.3850	0.2792	0.7773	0.135*	
H9C	0.2770	0.2957	0.6562	0.135*	
C10	0.2876 (3)	0.13132 (17)	0.8501 (2)	0.0807 (7)	
H10A	0.2722	0.1871	0.8782	0.097*	
H10B	0.2170	0.0967	0.8824	0.097*	
C11	0.4301 (3)	0.1034 (3)	0.9102 (3)	0.1159 (11)	
H11A	0.4337	0.1059	1.0023	0.174*	
H11B	0.5008	0.1386	0.8820	0.174*	
H11C	0.4462	0.0478	0.8844	0.174*	
C12	0.1202 (2)	0.15932 (15)	0.6641 (2)	0.0728 (6)	
H12A	0.1101	0.2136	0.7003	0.087*	
H12B	0.0550	0.1231	0.7008	0.087*	
C13	0.0797 (3)	0.1640 (2)	0.5198 (3)	0.1107 (11)	
H13A	−0.0148	0.1837	0.5028	0.166*	
H13B	0.0863	0.1103	0.4828	0.166*	
H13C	0.1418	0.2009	0.4824	0.166*	
C14	0.2893 (3)	0.04401 (13)	0.6544 (2)	0.0742 (6)	
H14A	0.3847	0.0273	0.6828	0.089*	
H14B	0.2791	0.0457	0.5611	0.089*	
C15	0.1902 (4)	−0.02043 (18)	0.6972 (4)	0.1230 (13)	
H15A	0.2104	−0.0727	0.6607	0.185*	
H15B	0.0954	−0.0051	0.6684	0.185*	
H15C	0.2019	−0.0243	0.7894	0.185*	
O1	0.15893 (14)	1.07037 (7)	0.10032 (11)	0.0582 (4)	
H1A	0.2006	1.1118	0.1392	0.087*	
O2	0.33056 (16)	0.76395 (8)	0.46118 (13)	0.0680 (4)	
O3	0.22788 (15)	0.70078 (7)	0.28835 (14)	0.0638 (4)	
N1	0.26670 (15)	0.12929 (9)	0.70443 (14)	0.0503 (4)	
O1W	0.5320 (2)	0.14390 (11)	0.3390 (2)	0.0996 (7)	
H1WA	0.4672	0.1691	0.2931	0.149*	
H1WB	0.5733	0.1776	0.3931	0.149*	

### Atomic displacement parameters (Å^2^)

**Table:**

	*U*^11^	*U*^22^	*U*^33^	*U*^12^	*U*^13^	*U*^23^
C1	0.0476 (8)	0.0300 (7)	0.0427 (8)	−0.0005 (6)	0.0027 (6)	0.0021 (6)
C2	0.0564 (9)	0.0385 (8)	0.0393 (8)	−0.0032 (7)	−0.0053 (7)	0.0000 (6)
C3	0.0492 (8)	0.0299 (7)	0.0451 (8)	−0.0055 (6)	0.0000 (6)	−0.0037 (6)
C4	0.0400 (7)	0.0301 (7)	0.0430 (8)	−0.0015 (5)	0.0034 (6)	0.0004 (6)
C5	0.0515 (9)	0.0348 (7)	0.0395 (8)	−0.0012 (6)	−0.0033 (6)	−0.0005 (6)
C6	0.0562 (9)	0.0284 (7)	0.0434 (8)	−0.0030 (6)	−0.0008 (7)	−0.0045 (6)
C7	0.0498 (9)	0.0318 (7)	0.0542 (9)	−0.0047 (6)	−0.0030 (7)	0.0046 (6)
C8	0.0608 (11)	0.0550 (10)	0.0710 (12)	−0.0007 (8)	0.0169 (9)	0.0034 (9)
C9	0.107 (2)	0.0529 (13)	0.109 (2)	−0.0090 (12)	0.0082 (15)	−0.0034 (12)
C10	0.0904 (16)	0.0989 (18)	0.0554 (12)	0.0158 (13)	0.0194 (11)	0.0025 (11)
C11	0.107 (2)	0.166 (3)	0.0718 (16)	0.019 (2)	−0.0044 (14)	0.0254 (19)
C12	0.0571 (11)	0.0777 (15)	0.0860 (15)	0.0130 (10)	0.0190 (10)	0.0032 (11)
C13	0.0775 (17)	0.160 (3)	0.0901 (19)	0.0169 (18)	−0.0113 (14)	0.0174 (19)
C14	0.0847 (15)	0.0489 (11)	0.0935 (16)	0.0037 (10)	0.0303 (12)	−0.0080 (10)
C15	0.129 (3)	0.0585 (15)	0.190 (4)	−0.0202 (16)	0.057 (3)	−0.0009 (18)
O1	0.0822 (9)	0.0318 (6)	0.0553 (7)	−0.0057 (5)	−0.0164 (6)	0.0071 (5)
O2	0.0993 (11)	0.0417 (7)	0.0567 (8)	−0.0127 (6)	−0.0194 (7)	0.0118 (6)
O3	0.0779 (9)	0.0287 (6)	0.0770 (9)	−0.0041 (5)	−0.0254 (7)	0.0012 (5)
N1	0.0538 (8)	0.0479 (8)	0.0519 (8)	0.0078 (6)	0.0181 (6)	−0.0004 (6)
O1W	0.0941 (12)	0.0703 (11)	0.1225 (15)	0.0175 (9)	−0.0410 (11)	−0.0246 (10)

### Geometric parameters (Å, °)

**Table:**

C1—O1	1.3515 (17)	C10—H10A	0.9700
C1—C6	1.389 (2)	C10—H10B	0.9700
C1—C2	1.392 (2)	C11—H11A	0.9600
C2—C3	1.379 (2)	C11—H11B	0.9600
C2—H2A	0.9300	C11—H11C	0.9600
C3—C4	1.392 (2)	C12—N1	1.505 (2)
C3—H3A	0.9300	C12—C13	1.516 (4)
C4—C5	1.389 (2)	C12—H12A	0.9700
C4—C7	1.505 (2)	C12—H12B	0.9700
C5—C6	1.381 (2)	C13—H13A	0.9600
C5—H5A	0.9300	C13—H13B	0.9600
C6—H6A	0.9300	C13—H13C	0.9600
C7—O2	1.241 (2)	C14—N1	1.506 (2)
C7—O3	1.2658 (19)	C14—C15	1.516 (4)
C8—C9	1.505 (3)	C14—H14A	0.9700
C8—N1	1.526 (2)	C14—H14B	0.9700
C8—H8A	0.9700	C15—H15A	0.9600
C8—H8B	0.9700	C15—H15B	0.9600
C9—H9A	0.9600	C15—H15C	0.9600
C9—H9B	0.9600	O1—H1A	0.8614
C9—H9C	0.9600	O1W—H1WA	0.8477
C10—C11	1.511 (4)	O1W—H1WB	0.8531
C10—N1	1.513 (3)		
			
O1—C1—C6	123.17 (13)	C10—C11—H11A	109.5
O1—C1—C2	117.58 (13)	C10—C11—H11B	109.5
C6—C1—C2	119.25 (13)	H11A—C11—H11B	109.5
C3—C2—C1	120.01 (13)	C10—C11—H11C	109.5
C3—C2—H2A	120.0	H11A—C11—H11C	109.5
C1—C2—H2A	120.0	H11B—C11—H11C	109.5
C2—C3—C4	121.45 (13)	N1—C12—C13	115.08 (19)
C2—C3—H3A	119.3	N1—C12—H12A	108.5
C4—C3—H3A	119.3	C13—C12—H12A	108.5
C5—C4—C3	117.73 (13)	N1—C12—H12B	108.5
C5—C4—C7	120.89 (13)	C13—C12—H12B	108.5
C3—C4—C7	121.38 (13)	H12A—C12—H12B	107.5
C6—C5—C4	121.60 (13)	C12—C13—H13A	109.5
C6—C5—H5A	119.2	C12—C13—H13B	109.5
C4—C5—H5A	119.2	H13A—C13—H13B	109.5
C5—C6—C1	119.96 (13)	C12—C13—H13C	109.5
C5—C6—H6A	120.0	H13A—C13—H13C	109.5
C1—C6—H6A	120.0	H13B—C13—H13C	109.5
O2—C7—O3	123.84 (14)	N1—C14—C15	114.5 (2)
O2—C7—C4	118.63 (13)	N1—C14—H14A	108.6
O3—C7—C4	117.51 (14)	C15—C14—H14A	108.6
C9—C8—N1	115.27 (19)	N1—C14—H14B	108.6
C9—C8—H8A	108.5	C15—C14—H14B	108.6
N1—C8—H8A	108.5	H14A—C14—H14B	107.6
C9—C8—H8B	108.5	C14—C15—H15A	109.5
N1—C8—H8B	108.5	C14—C15—H15B	109.5
H8A—C8—H8B	107.5	H15A—C15—H15B	109.5
C8—C9—H9A	109.5	C14—C15—H15C	109.5
C8—C9—H9B	109.5	H15A—C15—H15C	109.5
H9A—C9—H9B	109.5	H15B—C15—H15C	109.5
C8—C9—H9C	109.5	C1—O1—H1A	112.5
H9A—C9—H9C	109.5	C12—N1—C14	111.59 (17)
H9B—C9—H9C	109.5	C12—N1—C10	106.87 (15)
C11—C10—N1	115.1 (2)	C14—N1—C10	111.14 (17)
C11—C10—H10A	108.5	C12—N1—C8	110.52 (15)
N1—C10—H10A	108.5	C14—N1—C8	106.31 (14)
C11—C10—H10B	108.5	C10—N1—C8	110.46 (17)
N1—C10—H10B	108.5	H1WA—O1W—H1WB	108.8
H10A—C10—H10B	107.5		
			
O1—C1—C2—C3	−179.51 (15)	C3—C4—C7—O3	2.3 (2)
C6—C1—C2—C3	−0.1 (3)	C13—C12—N1—C14	−59.8 (3)
C1—C2—C3—C4	0.9 (3)	C13—C12—N1—C10	178.5 (2)
C2—C3—C4—C5	−0.8 (2)	C13—C12—N1—C8	58.3 (3)
C2—C3—C4—C7	179.84 (15)	C15—C14—N1—C12	−58.3 (3)
C3—C4—C5—C6	−0.1 (2)	C15—C14—N1—C10	60.9 (3)
C7—C4—C5—C6	179.22 (15)	C15—C14—N1—C8	−178.9 (2)
C4—C5—C6—C1	1.0 (3)	C11—C10—N1—C12	−179.8 (2)
O1—C1—C6—C5	178.56 (15)	C11—C10—N1—C14	58.2 (3)
C2—C1—C6—C5	−0.9 (2)	C11—C10—N1—C8	−59.5 (3)
C5—C4—C7—O2	2.0 (2)	C9—C8—N1—C12	57.8 (2)
C3—C4—C7—O2	−178.72 (16)	C9—C8—N1—C14	179.1 (2)
C5—C4—C7—O3	−176.97 (16)	C9—C8—N1—C10	−60.3 (2)

### Hydrogen-bond geometry (Å, °)

**Table:**

*D*—H···*A*	*D*—H	H···*A*	*D*···*A*	*D*—H···*A*
O1—H1A···O3^i^	0.86	1.74	2.5984 (16)	175
O1W—H1WA···O3^ii^	0.85	2.04	2.850 (2)	161
O1W—H1WB···O2^iii^	0.85	1.94	2.781 (2)	169

Symmetry codes: (i) −*x*+1/2, *y*+1/2, −*z*+1/2; (ii) −*x*+1/2, *y*−1/2, −*z*+1/2; (iii) −*x*+1, −*y*+1, −*z*+1.
